# Evolutionary Signals in Coronaviral Structural Proteins Suggest Possible Complex Mechanisms of Post-Translational Regulation in SARS-CoV-2 Virus

**DOI:** 10.3390/v14112469

**Published:** 2022-11-08

**Authors:** Ramiro Garza-Domínguez, Francisco Torres-Quiroz

**Affiliations:** Departamento de Bioquímica y Biología Estructural, División de Ciencia Básica, Instituto de Fisiología Celular, Universidad Nacional Autónoma de México, Mexico City 04510, Mexico

**Keywords:** post-translational modifications, coronaviruses, SARS-CoV-2, evolution, crosstalk

## Abstract

Post-translational regulation of proteins has emerged as a central topic of research in the field of functional proteomics. Post-translational modifications (PTMs) dynamically control the activities of proteins and are involved in a wide range of biological processes. Crosstalk between different types of PTMs represents a key mechanism of regulation and signaling. Due to the current pandemic of the novel and dangerous SARS-CoV-2 (Severe Acute Respiratory Syndrome Coronavirus 2) virus, here we present an in silico analysis of different types of PTMs in structural proteins of coronaviruses. A dataset of PTM sites was studied at three levels: conservation analysis, mutational analysis and crosstalk analysis. We identified two sets of PTMs which could have important functional roles in the regulation of the structural proteins of coronaviruses. Additionally, we found seven interesting signals of potential crosstalk events. These results reveal a higher level of complexity in the mechanisms of post-translational regulation of coronaviral proteins and provide new insights into the adaptation process of the SARS-CoV-2 virus.

## 1. Introduction

Recent studies in the field of functional proteomics have demonstrated that post-translational modifications (PTMs) represent the major level of protein regulation [1,2,3,4,5,6]. PTMs regulate the function of proteins acting as molecular switches and modulate protein structure controlling the interaction of proteins with other biomolecules. There are hundreds of different types of PTMs ranging from the covalent conjugation of basic functional groups and small polypeptides to structural changes including disulfide bond formation and proteolytic cleavage [1]. From an evolutionary point of view, different facts related to PTM conservation have been reported. For instance, it has been observed that residues susceptible to multiple PTMs exhibit greater conservation when compared to residues with a single PTM [7]. In another study, it was identified that highly conserved phosphorylation regions within domain families are often found near catalytic residues [5]. Additionally, it has been suggested that conservation signals associated with PTMs in intrinsically disordered regions have important roles in signaling and regulation [3]. From a functional perspective, it is known that different combinations of PTMs on proteins create intricate programs of control and regulation, and the crosstalk between different types of PTMs is now emerging as one of the central topics in proteomics research [1,8,9,10].

Given the functional importance of PTMs and due to the current pandemic of the novel Severe Acute Respiratory Syndrome Coronavirus 2 (SARS-CoV-2), the causative agent of the coronavirus disease 2019 (COVID-19), in this study, we present an analysis of different types of PTMs in the structural proteins of coronaviruses with an emphasis on the SARS-CoV-2 virus. The *Coronaviridae* family in the order *Nidovirales* is a group of enveloped RNA viruses which infect vertebrates, mainly birds and mammals [11,12]. Virions are spherical and decorated with large surface projections constituted by the spike glycoprotein. The genome is a linear, positive sense and non-segmented ssRNA molecule. The species in the family *Coronaviridae* share four structural proteins with the following characteristics [12]: The nucleocapsid protein (N) is involved in the packaging of the viral RNA genome into a helical ribonucleocapsid and plays a role during virion assembly. The envelope protein (E) is the smallest of the four structural proteins and exhibits ion channel activity. The membrane protein (M) is the most abundant in the virion and plays a central role in the formation of the viral particle. Finally, the spike protein (S) attaches the virion to the cell membrane and initiates the infection. Coronaviruses cause respiratory and gastrointestinal infections, and in humans these diseases range from common colds to severe respiratory infections with high mortality rates [12]. In the past two decades, two coronaviruses have caused important outbreaks, Severe Acute Respiratory Syndrome Coronavirus (SARS-CoV) and Middle East Respiratory Syndrome Coronavirus (MERS-CoV). SARS-CoV-2 is the third most dangerous coronavirus to acquire infectivity in humans; it has infected over 600 million people worldwide, and is responsible for more than 6 million deaths as of September 2022 [13], highlighting the importance of understanding the molecular properties and evolutionary dynamics of this virus.

The main objective of this study was to identify evolutionary signals on PTM sites of coronaviral structural proteins that suggest important functional roles. We compiled a dataset of 142 PTMs across 38 coronavirus species. For all coronaviral structural proteins, this study was performed at three levels: conservation analysis, mutational analysis and crosstalk analysis. First, multiple sequence alignments of different species were generated and the PTMs were mapped onto the alignments; then, we selected the PTM sites with the highest degree of conservation. In the second level, we considered a database of amino acid mutations on the SARS-CoV-2 virus and searched for the PTM sites with the lowest mutation frequency. Finally, considering the results obtained in the conservation and mutational analyses, we inspected the multiple sequence alignments in order to identify signals of possible crosstalk events between different types of PTMs. Altogether, this study identifies 52 PTM sites that could have important functional roles and nine signals with the potential to represent crosstalk mechanisms. The results obtained provide new insights into the post-translational regulation of coronaviruses and contribute to the understanding of the ongoing adaptation of the SARS-CoV-2 virus.

## 2. Materials and Methods

### 2.1. Coronavirus Species

According to the International Committee on Taxonomy of Viruses (ICTV), the taxonomic structure of the family *Coronaviridae* is divided into three subfamilies: *Letovirinae*, *Orthocoronavirinae* and *Pitovirinae* [11]. The *Orthocoronavirinae* subfamily is further subdivided into four genera: *Alphacoronavirus*, *Betacoronavirus*, *Gammacoronavirus* and *Deltacoronavirus*. In the latest version of the Master Species List published by the ICTV (2021.v1), there are 54 recognized coronavirus species [11]. The SARS-CoV-2 virus is not yet on the list. For this study, we considered the SARS-CoV-2 virus and coronavirus species recognized by the ICTV with a complete genome available in the NCBI RefSeq database. We found information about 38 species including the SARS-CoV-2 virus, all belonging to the *Orthocoronavirinae* subfamily. We considered the Severe Acute Respiratory Syndrome Coronavirus 2 isolate Wuhan-Hu-1 sequence, since this is the reference sequence of this species according to the NCBI RefSeq information. The accession numbers, genera, subgenera and species names of the 38 coronaviruses are listed in Supplementary Table S1.

### 2.2. Protein Sequences

The amino acid sequences related to the four structural proteins (N, E, M and S) of the 38 coronavirus species were collected from the NCBI Protein database.

### 2.3. PTM Dataset

A number of databases are available for PTM sites; however, we only found information about coronaviruses in three databases: PhosphoSitePlus [14], BioGRID [15] and UniProt [16]. Many of the PTMs reported in these databases were inferred by sequence similarity; nevertheless, we chose only PTMs with experimental evidence. We compiled a dataset of PTM sites from these databases and mainly from published studies [17,18,19,20,21,22,23,24,25,26,27,28,29,30,31,32,33,34,35,36,37,38,39,40,41,42,43,44]. This resulted in a total of 142 PTMs sites of 6 different types: 57 phosphorylation sites, 51 N-glycosylation sites, 12 O-glycosylation sites, 16 palmitoylation sites, 5 methylation sites and 1 sumoylation site. The complete dataset of the PTMs is shown in Supplementary Table S2. The PTM sites are listed according to the species, protein and PTM type, and the corresponding references are shown in the last column.

### 2.4. Multiple Sequence Alignments

Using protein sequences in FASTA format as inputs, the multiple sequence alignments were generated on the UniProt server with the Clustal Omega program [16]. The outputs were further processed with custom software.

### 2.5. PTM Mapping and Conservation

For each type of coronaviral structural protein, the PTMs were mapped to the multiple sequence alignments. Due to the lack of PTM data available, the following assumption was made: all residues in the same column that were identical to the amino acid in a PTM site were considered PTM sites; this is called PTM propagation [45]. The strategy of propagating the PTM signal from validated modified sites through orthologous proteins was proposed by Minguez et al. They found that propagated PTMs exhibit a good overlap with known PTMs and propose that this strategy is an interesting indicator of the PTMs’ reliability [45]. For the PTM sites related to the SARS-CoV-2 virus, the degree of conservation was calculated. We define the degree of conservation as the number of residues in the same column that are identical to the amino acid in a PTM site of the SARS-CoV-2 sequence. The degree of conservation is expressed as a percentage. In order to make general propositions about the PTM sites, we only considered PTM sites located in columns without gaps. In the context of computational genomics and phylogenetics, it is common practice not to consider positions in columns with gaps because these sites are ambiguous. On the other hand, the positions in columns without gaps represents homologous sites across the species [46,47].

To illustrate these concepts, let us consider the dataset of 4 PTM sites shown in Supplementary Figure S1A. Mapping the PTMs to the multiple sequence alignment consists of identifying the positions of the PTMs on the alignment; for example, the phosphorylation at serine 197 in the Murine coronavirus sequence corresponds to the position 229 in the alignment (Supplementary Figure S1B). Propagating a PTM refers to inferring that the modification exists in all species in which the residue is the same as the amino acid in the PTM site (Supplementary Figure S1C). It can be seen that the two phosphorylations in the Severe Acute Respiratory Syndrome-related coronavirus (SARS-CoV) sequence are propagated to SARS-CoV-2. However, the phosphorylation in the Murine coronavirus sequence is not propagated to SARS-CoV-2 because there is a glicine in that position in the SARS-CoV-2 sequence (Supplementary Figure S1C). After propagating the signal, there are 3 PTM sites related to SARS-CoV-2. Nevertheless, only 2 sites are located in columns without gaps; therefore, the degree of conservation is calculated only for these two sites, which correspond to positions 176 and 184 in the SARS-CoV-2 sequence (Supplementary Figure S1D).

### 2.6. Analysis of Mutations

The database CoV-GLUE (update: November 2021) was considered for this analysis [48]. This database is based on the GISAID repository which maintains information about millions of SARS-CoV-2 genome sequences. The database CoV-GLUE stores information about amino acid replacements observed from the ongoing COVID-19 pandemic. In the case of the four structural proteins, the mutations reported in this database are: 6869 for the N protein, 1211 for the E protein, 3064 for the M protein and 19,797 for the S protein [48]. Different substitutions have been observed in all the residues of the structural proteins and each substitution has a certain frequency associated with it. Based on the frequencies of the different substitutions reported for a given PTM site, the sum of frequencies was calculated and normalized with respect to the higher value; we call this the mutation frequency for the PTM site.

Consider the three PTM sites related to the SARS-CoV-2 virus shown in Supplementary Figure S1C; since the analysis of mutations does not depend on the species alignment, the mutation frequency was calculated for all three sites. For example, in position 176, nine different substitutions were observed, each with its respective frequency; the sum of the frequencies is 77, and by dividing it by the higher value, 1459 in this illustrative example, the mutation frequency for this PTM site is 0.0527 (Supplementary Figure S1E).

### 2.7. Fuzzy Sets

In order to select the PTM sites with the highest degree of conservation and the lowest mutation frequency, we used the approach of fuzzy sets. A fuzzy set has indistinct boundaries and is used to measure to what degree something is a member of a particular set [49,50]. The degree of membership tells us how compatible a particular element is with the idea that the set represents. A fuzzy set is defined by a membership function; the triangular function is the most common, the horizontal axis represents the members of the set and the vertical axis represents the degree of membership in the set [49,50]. Given a numerical variable ranging from the lowest value to the highest value, a linguistic variable can be defined with a group of overlapping fuzzy sets underlying the complete range of numeric values; this is a common practice in the context of fuzzy logic applications [49,50]. In our analysis, we defined linguistic variables based on five fuzzy sets. The fuzzy sets were labeled with the linguistic values: “Very Low”, “Low”, “Mid”, “High” and “Very High”. The numeric values were mapped to the linguistic variables to calculate the degree of membership (a number between 0 and 1) of each value to the fuzzy sets; then, each value was assigned to the fuzzy set with the highest degree of membership.

As an example, consider the case of the nucleocapsid protein; there are 10 PTM sites located in columns without gaps on the alignment, so the degree of conservation was calculated for these 10 sites (Supplementary Figure S2A). These values represent a numerical variable ranging from 7 to 73; then, we can define a linguistic variable with the group of five overlapping fuzzy sets (Supplementary Figure S2B). Now, let us take the first element, the PTM site N47 with a degree of conservation of 21; this value has a degree of membership of 0.1515 to the fuzzy set “Very Low”, 0.8484 to the fuzzy set “Low” and 0 to the rest of the sets (Supplementary Figure S2B). Therefore, the linguistic value of 21 is “Low”. This procedure was applied to all PTM sites (Supplementary Figure S2C). As we were interested in identifying the PTM sites with the highest degree of conservation, we considered the sites with linguistic values “High” and “Very High” as sites of interest; in this case, there are two PTM sites of interest, K61 and S184 (Supplementary Figure S2C). If we had used crisp intervals instead of fuzzy sets, as shown in Supplementary Figure S2D, and we would have decided to choose the sites in intervals 3 and 4 as the PTM sites of interest, we can see that there are 3 elements in interval 3. These 3 elements correspond to the sites K61, T148 and S176 with conservation values of 52, 47 and 42, respectively. The last two values do not represent the idea of “High” well; fuzzy sets capture the intention instead of the mechanics of the selection process, and this intentionality improves the semantics of the selection [49,50].

The values of the mutation frequency for the 32 PTM sites in the nucleocapsid protein are shown in Supplementary Figure S2E. In this case, it is more difficult to decide between the idea of low and high, and fuzzy sets facilitate this selection process. Since we were interested in identifying the PTM sites with the lowest mutation frequency and with the intention to remove the outlier values, we considered only PTM sites with a mutation frequency lower than 0.06. Additionally, for the analysis of mutations, we removed the PTM sites selected as sites of interest in the conservation analysis. Supplementary Figure S2F shows the results of the selection process based on fuzzy sets. Contrary to the conservation analysis, in the mutational analysis, the PTM sites of interest are those with linguistic values “Very Low” and “Low”.

In an effort to perform all the procedures described in Section 2.5, Section 2.6 and Section 2.7, a custom program was developed, and this program was written in the C++ programming language. All code and data needed for the calculations are accessible through the url https://github.com/RamiroUNAM/CoronaPTM (accessed on 5 October 2022).

### 2.8. Kinase Prediction

For certain phosphorylation sites of interest, the NetPhos 3.1 server was used to predict the possible protein kinases responsible for the modification of those sites [51].

## 3. Results and Discussion

**Figure 1:**
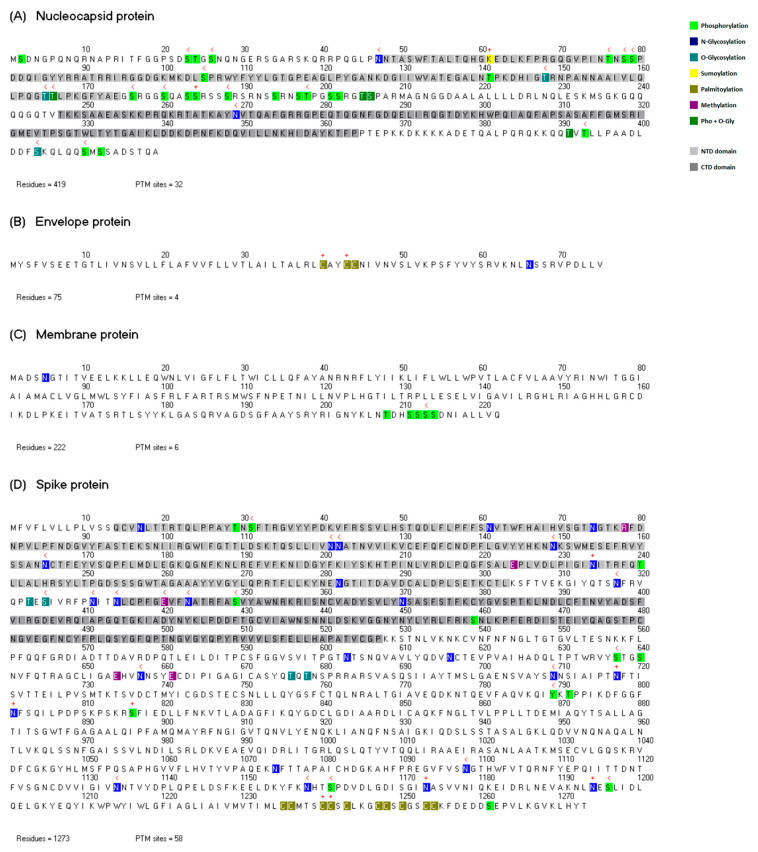
Amino acid sequences of the 4 structural proteins of SARS-CoV-2 virus and their PTM sites. The functional domains and the PTM sites are represented in different colors as indicated at the top right of the Figure. The nucleocapsid protein contains 4 multiPTM sites (depicted in dark green), which are sites with different types of PTMs observed at the same position, in this case phosphorylations and O-glycosylations. The residues labeled with a “+” symbol correspond to the PTM sites with the highest degree of conservation (see Section 3.1), and the residues labeled with a “<” symbol correspond to the PTM sites with the lowest mutation frequency (see Section 3.2).

As mentioned previously, this study was performed at three levels: conservation analysis, mutational analysis and crosstalk analysis. We describe each case in turn. Figure 1 shows the amino acid sequences of the four structural proteins of the SARS-CoV-2 virus and their PTM sites. There are 100 PTM sites in the four proteins. The nucleocapsid protein contains two functional domains, the N-terminal domain (NTD) and the C-terminal domain (CTD). The NTD is involved in RNA binding while the CTD is related to protein dimerization. The spike protein also contains two functional domains: the NTD contributes to the S trimer interface, whereas the CTD serves as the receptor-binding domain (RBD). The functional domains and the PTM sites are represented in different colors as indicated in Figure 1.

### 3.1. Conservation Analysis

**Figure 2:**
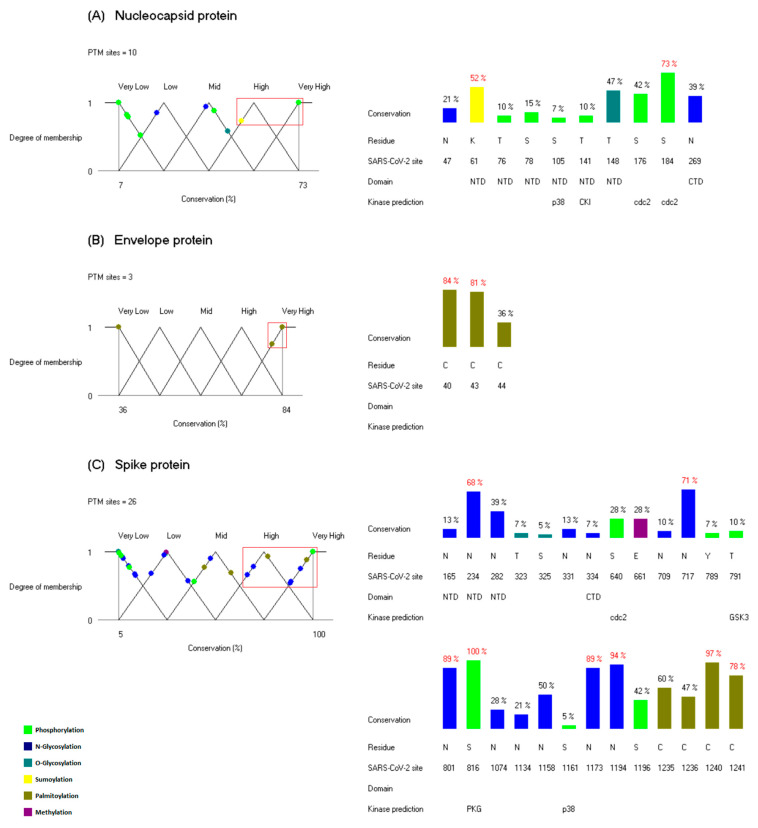
PTM sites with the highest degree of conservation in the coronaviral structural proteins. For each structural protein, (**A**) Nucleocapsid, (**B**) Envelope and (**C**) Spike. A representation of the linguistic variable related to the degree of conservation is shown. The points assigned to the fuzzy sets “High” and “Very High” (framed in a red rectangle) were selected as sites of interest. The histograms on the right indicate the degree of conservation of the PTM sites, and the values in red correspond to the PTM sites of interest. In the histograms, the numbers at the top of the bars represent the degree of conservation expressed in percentages, and the numbers below indicate the residue, the position in the SARS-CoV-2 sequence, the functional domain in which the residue is located and the kinase prediction if the PTM site corresponds to a phosphorylation. The distinct types of PTMs are represented in different colors as indicated at the bottom left of the Figure.

Multiple sequence alignments based on the 38 coronaviral species were generated for the four types of structural proteins N, E, M and S. The PTMs were mapped and propagated onto the alignments (Supplementary Figures S3–S6), and for the PTM sites related to the SARS-CoV-2 virus, the degree of conservation was calculated, and we only considered the PTM sites located in columns without gaps. The conservation degree values were mapped to the linguistic variables and the points assigned to the fuzzy sets “High” and “Very High” were selected as the sites of interest. Figure 2 shows the representation of the linguistic variables, and the histograms below indicate the degree of conservation of the PTM sites of interest. Of the 100 PTM sites in the four structural proteins, only 40 sites are located in columns without gaps on the alignments.

There are two PTM sites with a high degree of conservation among the coronaviruses in the N protein: one sumoylation site located in the NTD, and one phosphorylation site between the two functional domains (Figure 1A and Figure 2A). The lysine at position 61 corresponds to a sumoylation site. This modification was first identified at lysine 62 in the nucleocapsid protein of the SARS-CoV virus, and it was demonstrated that the sumoylation of the N protein promotes its homo-oligomerization and participates in the interference of host cell division [30].

This lysine residue was conserved in all members of the genera *Betacoronavirus* and *Deltacoronavirus*. This was an unexpected correlation since the genus *Deltacoronavirus* is the most phylogenetically distant from the genus *Betacoronavirus* [12]. This observation points to the possibility that the sumoylation event could exist in some species of these two groups and performs the same functionalities as the SARS-CoV virus. The phosphorylation site S184 is located between the two functional domains; this region is usually called the SR-rich domain and it corresponds to an intrinsically disordered region. It is known that phosphorylation events often occur in this region in different coronaviruses and that they play key roles in the regulation of the N protein [52]. There are 12 phosphorylation sites in the SR-rich region of the SARS-CoV-2 virus according to our PTM dataset (Figure 1A). Given that the residue S184 is the only one site in this region located in a column without gaps and with a high conservation degree, we propose that this phosphorylation must have an important functional role among the coronaviruses. For this phosphorylation site, the kinase predicted is cdc2, and this kinase could be a potential therapeutic target. According to UniProt information, cdc2 is also called cyclin-dependent kinase 1 (CDK1) and plays an important role in the control of the cell cycle [16].

In the case of the envelope protein, there are two highly conserved cysteines related to palmitoylation events. These palmitoylated cysteines have been studied in the Murine coronavirus and SARS-CoV virus. It was demonstrated that palmitic acid modifications are essential for virion assembly and are important for protein stability in the Murine coronavirus [18,19]. In the SARS-CoV virus, some associations of palmitoylation events with subcellular trafficking and lipid rafts have been observed [27]. The high degree of conservation of the residues C40 and C43 supports the functional significance of these PTM sites in the species of the family *Coronaviridae*.

There is only one PTM site located in a column without gaps on the alignment of coronaviral membrane proteins (not shown in Figure 2); this site has a low degree of conservation and may not play an essential role for the coronaviruses in general.

Finally, in the spike protein, there are 8 PTM sites with a high degree of conservation, 5 N-glycosylation sites, 2 palmitoylation sites and 1 phosphorylation site (Figure 2C). It is well known that the spike protein of coronaviruses is highly glycosylated and that these glycosylations play a key role in protein folding and antibody recognition [52]. The N-glycosylations in the spike protein of the SARS-CoV-2 virus have been reported independently in different recent publications [31,32,33,34,35,36]. There is one conserved N-glycosylation site in the NTD, and the other four highly conserved N-glycosylation sites are located downstream of the functional domains. It has been proposed that the N-glycosylations at the sites N1173 and N1194, which are located in the stalk of the protein, protect important hinges from antibody binding, and this gives to the S protein the flexibility to scan the host cell surface [53,54]. The high degree of conservation of these PTM sites is in agreement with this important functionality of the spike protein. The glycosylation events in N234 and N717 were previously identified in their homologous residues N227 and N699 of the SARS-CoV virus [23]. It was demonstrated that the SARS-CoV virus uses the lectins DC-SIGN and L-SIGN as alternative receptors to ACE2 and certain N-glycosylation sites are required for virus entry; among these sites are the residues N227 and N699 [23]. As can be seen in Figure 2C, there is a highly conserved phosphorylation site, S816. The phosphorylations in the spike protein were detected by Davidson et al. [39], and this type of modification had not been identified before in the S protein of coronaviruses. Interestingly, the residue S816, which is a surface site [39], has a degree of conservation of 100%, and this is the only PTM site in the four structural proteins with this property. The kinase predicted for this site is PKG; this kinase is also called cGMP-dependent protein kinase 1 and acts as a mediator of the nitric oxide signaling pathway [16]. This molecule could be another therapeutic target candidate for coronavirus infections.

Of the 10 palmitoylation sites located in the C-terminal region of the S protein (Figure 1D), only the sites C1240 and C1242 are highly conserved (Figure 2C). In the infected cell, massive lipidation controls spike biogenesis and allows the formation of viruses with enhanced fusion capacity [38]. Additionally, it has been suggested that palmitoylation events in the S protein modulate the membrane curvature, and this facilitates the fusion with the host cell [54]. Therefore, the PTM sites C1240 and C1242 could be key in these functions for coronaviruses.

Altogether, the conservation analysis identified 12 PTM sites highly conserved among the members of the *Coronaviridae* family. Several functional correlations related to some of these sites were observed in different coronaviral species. We propose that these 12 residues could be the most important PTM sites in the regulation of the structural proteins of coronaviruses in general.

### 3.2. Mutational Analysis

**Figure 3:**
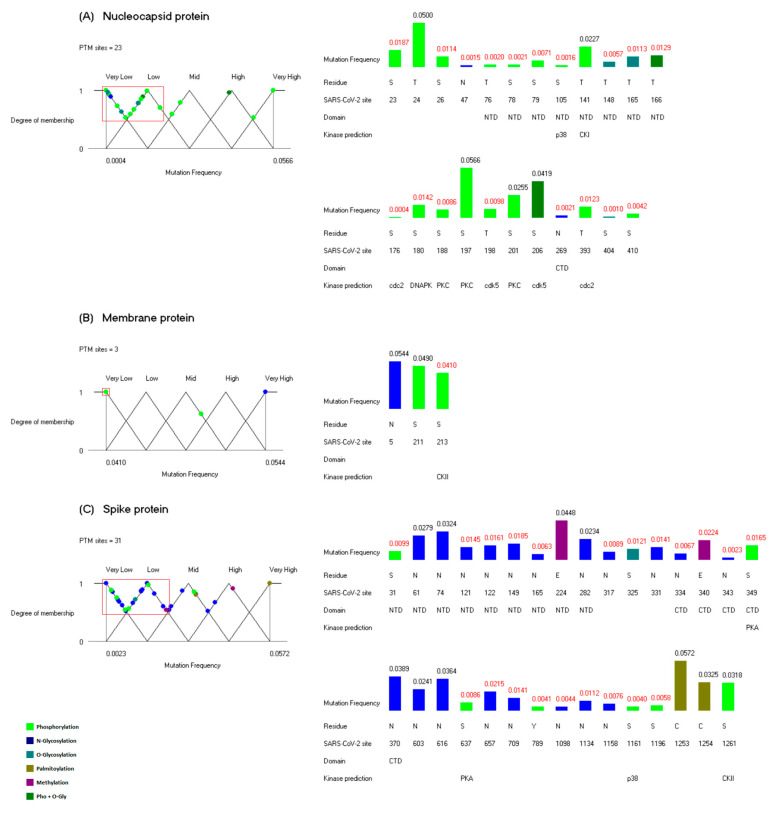
PTM sites with the lowest mutation frequency in the SARS-CoV-2 structural proteins. For each structural protein, (**A**) Nucleocapsid, (**B**) Envelope and (**C**) Spike. A representation of the linguistic variable related to the mutation frequency is shown. The points assigned to the fuzzy sets “Very Low” and “Low” (framed in a red rectangle) were selected as sites of interest. The histograms on the right indicate the mutation frequency of the PTM sites, and the values in red correspond to the PTM sites of interest. In the histograms, the numbers at the top of the bars represent the mutation frequency, and the numbers below indicate the residue, the position in the SARS-CoV-2 sequence, the functional domain in which the residue is located and the kinase prediction if the PTM site corresponds to a phosphorylation. The distinct types of PTMs are represented in different colors as indicated at the bottom left of the Figure.

Based on the information of the CoV-GLUE database, for each PTM site, the different substitutions and their frequencies observed from the ongoing pandemic were retrieved. The mutation frequencies of the PTM sites were calculated and subjected to further analysis (see Section 2.6). These frequencies were in the following ranges: 0.0004 to 1 for the nucleocapsid protein, 0.1453 to 1 for the envelope protein, 0.0410 to 1 for the membrane protein and 0.0016 to 1 for the spike protein. From the point of view of the PTM sites, it is clear that the nucleocapsid protein presents the highest mutational pressure followed by the S, M and E proteins. We considered only PTM sites with a mutation frequency lower than 0.06 that were not selected for the conservation analysis, and there are 57 sites with these properties (Figure 3). The mutation frequency values were mapped to linguistic variables, and in this case, the points assigned to the fuzzy sets “Very Low” and “Low” were selected as sites of interest. In Figure 3, the linguistic variables are depicted, and the histograms indicate the mutation frequency of the PTM sites. Of the 100 PTM sites in the four structural proteins, there are 40 PTM sites with a low mutation frequency (Figure 3); we propose that these 40 PTM sites could play important functional roles, specifically in the SARS-CoV-2 virus.

There are 18 of these sites in the nucleocapsid protein (see Figure 3A), and it has been hypothesized that the phosphorylations in the NTD affect the surface charge of this region, and thus modulate the function of the N protein [40]. With the exception of the site T141, the rest of the phosphorylations in the NTD must be critical in this modulation process, mainly the site S105 which has the lowest mutation frequency. There are 8 phosphorylation sites of interest outside of the NTD, 2 in the N-terminal region, 4 in the SR-rich region and 2 in the C-terminal region (Figure 3A). Due to their low mutation frequency, these phosphorylation sites must be important for the regulation of the N protein. The kinase predicted for the sites S176 and T393 was cdc2 (CDK1), as was also predicted for the conserved residue S184, and the kinase predicted for the site S188 was PKC, as was also predicted for the highly conserved residue S816 of the spike protein. In the case of the residue S176, it was demonstrated that the phosphorylation of the homologous residue S177 in the SARS-CoV virus is regulated by the kinase GSK-3 [22]. Additionally, Hekman et al. confirmed that the residue S176 of the SARS-CoV-2 virus is phosphorylated by GSK-3 [41]. Taken together, in silico and in vitro results point to kinases cdc2 (CDK1), PKC and GSK-3 as potential therapeutic targets. The N-glycosylation and O-glycosylation sites were identified in a recent study directly on the nucleocapsid protein of the SARS-CoV-2 virus [42]. Glycosylation events in the N protein had not been described before in the coronaviruses and their functional roles are unknown. As shown in Figure 3A, the sites N47, T148, T165, N269 and S404 have a low mutation frequency, and this observation suggests that these glycosylations could have a role in the regulation of the N protein.

The mutation frequency of all the PTM sites in the envelope protein are greater than 0.06; therefore, there are no sites of interest in this protein from the point of view of our mutational analysis.

In the case of the membrane protein, there are three PTM sites with a mutation frequency lower than 0.06. The site S213 has the lowest value (Figure 3B). This site is clustered with the other four phosphorylated residues at the C-terminal region of the membrane protein (Figure 1C). It has been suggested that a negative charge in this region of the M protein could play a key functional role [40]. The notably low mutation frequency observed at S213 pinpoints this site as the most important of the five phosphorylated residues.

Finally, in the spike protein, there are 21 PTM sites of interest (Figure 3C). It was observed that the glycosylations at residues N165 and N343 are used by the S protein to camouflage the receptor binding sites [31]. The glycosylation event at N343 was first identified in its homologous residue N330 of the SARS-CoV virus [25]. It was demonstrated that this residue plays an important role in the interaction between the S protein and the lectin DC-SIGN [25]. The N-glycosylation at site N317 has not been identified in the SARS-CoV-2 virus; it was derived from PTM propagation. This latter N-glycosylation was detected and described in Avian coronavirus, a member of the genus *Gammacoronavirus* [43]. Nevertheless, the low mutation frequency of the residue N317 suggests that this is an important asparagine for the SARS-CoV-2 virus. In the case of the O-glycosylation at site S325, it has been proposed that this PTM could play a key role in the binding with ACE2 receptors [33]. There are six phosphorylation sites with a low mutation frequency in the spike protein. For the sites S349 and S637, the kinase predicted is PKA, whereas for the site S1161, the kinase predicted is p38; curiously, the same prediction was made for the site S105 of the N protein mentioned above (Figure 3A). PKA is also known as cAMP-dependent protein kinase and phosphorylates several substrates in the cell [16]. The kinase p38 is also called mitogen-activated protein kinase 11 and is an essential component of the MAP kinase signal transduction pathway [16]. Thus, these two kinases could be other interesting therapeutic target candidates. As for the phosphorylation residue Y789, it was suggested that this PTM site could be involved in the trimerization of the S protein [39]. This is the only phosphorylation event observed on a tyrosine residue in all the structural proteins. There are five methylation sites in the S protein (Figure 1D). Methylations in the spike protein were detected for the first time by Sun et al. [35], and their functional roles are unknown. The methylated residue E340 located in the CTD was selected as a site of interest, suggesting that this modification could be important for the S protein.

Collectively, the mutational analysis identified a second group of 40 PTM sites that, due to their low mutation frequency, could play important roles in the regulation of the structural proteins of the SARS-CoV-2 virus.

### 3.3. Crosstalk Analysis

**Figure 4:**
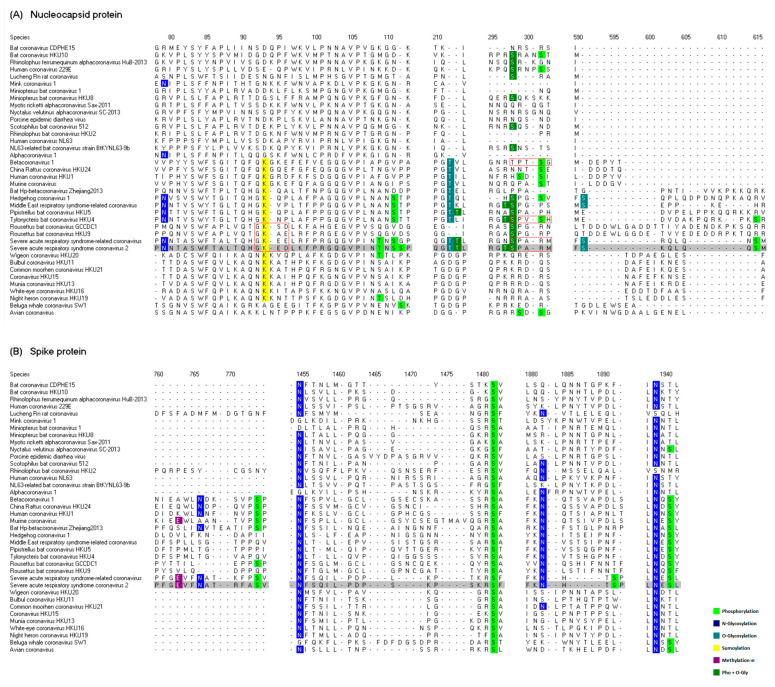
Crosstalk signals in the nucleocapsid and spike proteins. Eight regions of interest in the multiple sequence alignments of (**A**) Nucleocapsid and (**B**) Spike proteins are shown. The numbers at the top correspond to the positions on the alignments. The species names of the 38 coronaviruses are listed on the left side, and the row related to SARS-CoV-2 virus is highlighted in gray. The letters framed in a red rectangle correspond to sequence motifs related to specific PTM types. The PTM sites are represented in different colors as indicated at the bottom right of the Figure.

Hunter defines two general types of PTM crosstalk, positive and negative [9]. In positive crosstalk, one PTM acts as a signal for the addition or elimination of a second PTM, whereas in negative crosstalk, there is a direct competition of distinct PTMs for the same residue or one PTM can hinder the recognition site for a second PTM [9]. In our crosstalk analysis, the multiple sequence alignments were inspected in order to identify possible crosstalk events between different types of PTMs. We considered the high conservation degree, low mutation frequency and close proximity in the SARS-CoV-2 sequence as the parameters to evaluate different cases. We found nine signals of possible crosstalk events: five in the nucleocapsid protein and four in the spike protein. Figure 4 displays eight regions of interest in the multiple sequence alignments of N and S proteins.

In Figure 4A, it can be seen that four regions of interest related to the nucleocapsid protein, the first region ranging from positions 78 to 113, contains five PTM sites: 1 N-glycosylation site, 1 sumoylation site and 3 phosphorylation sites. The residue at position 93 on the alignment corresponds to the lysine 61 in the SARS-CoV-2 sequence and the phosphorylation sites at positions 109, 111 and 112 on the alignment correspond to the positions 76, 78 and 79 in the SARS-CoV-2 sequence. The sumoylation site K61 was selected as a site of interest in the conservation analysis. The three phosphorylation sites that are in close proximity to the sumoylation site have a low mutation frequency and were selected in the mutational analysis. These four PTM sites are located in the NTD of the N protein (see Figure 1A), and this observation invites us to consider a possible crosstalk between phosphorylation and sumoylation. A number of crosstalk events involving sumoylation and phosphorylation have been reported in different proteins. For example, it was demonstrated that sumoylation promotes the autophosphorylation of the protein PYK2 and then the two PTMs cooperate to activate the SRC-PYK2 complex [55]. In another study, it was observed that the transcriptional activator NF-E2 is phosphorylated by the kinases PKA and ERK1, enhancing the sumoylation of NF-E2, which leads to the activation of its transcriptional activity [56]. Additionally, it was proved that the sumoylation and triple serine phosphorylation of the oligodendrocyte transcription factor 2 (Olig2) are necessary for the antiapoptotic function of this protein [57]. Sumoylation often occurs at lysines located within the consensus sequence Ψ-K-X-E/D, where Ψ is any hydrophobic amino acid and X is any amino acid. As can be seen in Figure 4A, the lysines of four species fit this motif (letters framed in a red rectangle), and these correspond to the species *Rousettus bat coronavirus GCCDC1* and *Rousettus bat coronavirus HKU9* from the subgenus *Nobecovirus*. The other two species are *SARS-CoV* and *SARS-CoV-2* from the subgenus *Sarbecovirus*. Sumoylation events in other species cannot be ruled out since a number of sumoylations lacking the consensus motif have been detected [56,57]. Therefore, since the highly conserved sumoylation site K61 is located within the consensus motif and in close proximity to the phosphorylation sites T76, S78 and S79, which have a low mutation frequency, we point to this signal as a potential positive crosstalk event between phosphorylation and sumoylation.

There is a second signal of a positive crosstalk in this same region. Curiously, the N-glycosylated asparagine at position 79 and the phosphorylated serine at position 111 on the alignment, which correspond to the sites N47 and S78 in the SARS-CoV-2 sequence, are conserved exactly in all the species of the subgenera *Merbecovirus* and *Sarbecovirus* (see Figure 4A). The three dangerous human coronaviruses MERS-CoV, SARS-CoV and SARS-CoV-2 belong to these subgenera. Both sites N47 and S78 have a low mutation frequency and are in a relatively close proximity; nevertheless, the interplay between N-glycosylation and phosphorylation is poorly understood.

The other three regions of interest in the N protein contain signals of potential negative crosstalk events between phosphorylation and O-glycosylation. One of the first described and well-known mechanisms of negative crosstalk refers to the interplay between O-glycosylation and phosphorylation [58,59]. Both PTMs compete for the same serine or threonine residues, and this is known as “Reciprocal interplay” [58,59]. O-glycosylation can prevent phosphorylation at specific residues or adjacent sites. As mentioned previously, there are four multiPTM sites in the N protein: T166, T205, S206 and T391 (see Figure 1A). These sites in principle represent possible negative crosstalk events. The multiPTM site T166 (position 213 on the alignment) adjacent to the O-glycosylation site T165 (position 212 on the alignment) located in the second region of interest (Figure 4A) was selected in the mutational analysis. These two sites are conserved in the sarbecoviruses and are located in the NTD of the SARS-CoV-2 virus, suggesting a possible negative crosstalk in this domain.

It has been proposed that the proteins containing the sequence motif [S/T]P[V/A/T][S/T]X-p, where X-p represents any amino acid except proline, can be regulated by a negative crosstalk between phosphorylation and O-glycosylation [59]. In the third region of interest (Figure 4A), the multiPTM site S206 (position 298 on the alignment) shows some interesting properties that suggest the existence of a negative crosstalk in the species of the genus *Betacoronavirus*. Even though this site was not selected in the conservation or mutational analyses, this serine has a relatively high conservation degree, as it is conserved in eight species of the genus *Betacoronavirus* and in seven species of the genus *Alphacoronavirus*.

There is one threonine in column 298 related to the species *Betacoronavirus 1*; since threonine residues are susceptible to be phosphorylated and O-glycosylated, we considered this residue as a possible site involved in negative crosstalk. Interestingly, two species show an exact match to the sequence motif mentioned above: *Betacoronavirus 1* and *Tylonycteris bat coronavirus HKU4*. SARS-CoV and SARS-CoV-2 viruses differ in one amino acid from the motif (letters framed in a red rectangle in Figure 4A). Therefore, given that the PTM site S206 has a relatively high conservation degree and some species match the consensus motif, we propose this case as a potential negative crosstalk mechanism.

In the fourth region of interest, there are two PTM sites with a low mutation frequency: the O-glycosylated site S404 (position 591 on the alignment) and the phosphorylated site S410 (position 615 on the alignment). Since these two sites are separated only by five amino acids, they could be involved in a negative crosstalk event.

In the case of the spike protein, the four regions of interest range from residues 760–775, 1454–1483, 1880–1893 and 1938–1942 (Figure 4B). In the first region, there are three sites of three different types of PTMs located in very close proximity, and these sites correspond to the SARS-CoV-2 residues E340, N343 and S349. These three PTM sites are located in the CTD and are conserved in the two species of the subgenus *Sarbecovirus*. Despite their low conservation, the three sites have a low mutation frequency, specially the site N343 (see Figure 3C). The functional role of phosphorylation and methylation in the spike protein is unknown. To what extent these two types of PTMs influence the N-glycosylated sites in this important receptor-binding domain remains to be further investigated. Given the close proximity and low mutation frequency of these three PTM sites, we propose that this signal could be a potential crosstalk event specific to the sarbecoviruses in the CTD.

In the other three regions, there are two PTM sites in close proximity: a N-glycosylation site followed by a phosphorylation site. Contrary to the latter case, the two PTM sites in the second region located at positions 1455 and 1482 on the alignment have a very high conservation degree. These sites correspond to the residues N801 and S816 in the SARS-CoV-2 sequence and were selected as sites of interest in the conservation analysis (see Figure 2C). This is the strongest signal of a possible crosstalk in the coronaviral structural proteins.

In the third region, the two PTM sites correspond to the SARS-CoV-2 residues N1158 and S1161 and are separated only by two amino acids. The first site has a relatively high conservation degree and the two sites have a low mutation frequency (Figure 3C).

In the fourth region of interest, the N-glycosylation site at position 1939 on the alignment has a high degree of conservation. The other PTM site in this region, related to a phosphorylated serine at position 1941 on the alignment, has a relatively high conservation degree. Nevertheless, it can be appreciated in Figure 4B that the majority of the remaining residues in this column are threonines. Since the threonine residues are also susceptible to being phosphorylated, the conservation degree of the phosphorylation status is exactly the same as the N-glycosylation site. These two sites correspond to the residues N1194 and S1196 in the SARS-CoV-2 sequence. The first site has a high conservation degree (Figure 2C), the second site has a low mutation frequency (Figure 3D), and the two residues are separated only by one amino acid. This is another interesting signal of a possible crosstalk between phosphorylation and N-glycosylation.

Little is known about the interplay between N-glycosylation and phosphorylation; however, our results show five signals (one in the N protein and four in the S protein) of the possible crosstalk mechanisms in which these two PTMs are involved. Davidson et al. suggest that the phosphorylated residues could be considered as potential control points for spike conformational changes [39]. Since the N-glycosylation sites play a key role in protein folding, maybe the phosphorylation sites in close proximity contribute to this modulation process.

Finally, it can be seen that there are five methylation sites in the S protein (Figure 1D). Curiously, these five sites are in close proximity of N-glycosylation sites. Four methylated residues were not selected in our conservation and mutational analyses; however, this observation invites us to consider the possibility of the existence of crosstalk events between N-glycosylation and methylation. These issues remain to be further investigated.

## 4. Conclusions

In summary, we identified two sets of PTM sites which could have important functional roles. The first set is composed of 12 PTM sites with a high degree of conservation across the species of the family *Coronaviridae*. These sites could represent general mechanisms of regulation common to all coronaviruses. The second set contains 40 PTM sites with a low mutation frequency. These latter sites could be involved in strategies of regulation specific to the SARS-CoV-2 virus. Based on computational predictions, the kinases cdc2 or cyclin-dependent kinase 1, PKG or cGMP-dependent protein kinase 1, PKA or cAMP-dependent protein kinase and p38 or mitogen-activated protein kinase 11, could be possible therapeutic targets. Finally, we identified nine interesting signals of potential crosstalk events between different types of PTMs. The big picture of all PTM types in all structural proteins across all species of the family *Coronaviridae* (not considered in other studies) unveil a higher level of complexity related to the post-translational regulation mechanisms of coronavirus species. The observations presented in this study could be important for prioritizing experimental validations and for the exploration of new therapeutic targets involving different modification enzymes. Possible coronavirus outbreaks will continue to be a permanent health threat in the world, and the understanding of the molecular and evolutionary mechanisms of coronaviruses is critical to deal with the current and future epidemics. The results described in this work contribute to this direction and open the way to explore new ideas and techniques for the study of these important viruses.

## Data Availability

The data presented in this study are available in Supplementary Materials.

## Supplementary Materials

The following supporting information can be downloaded at https://www.mdpi.com/article/10.3390/v14112469/s1: Figure S1: PTM mapping, propagation, conservation and mutation frequency; Figure S2: Selection process based on fuzzy sets; Figure S3: Multiple sequence alignment related to the nucleocapsid protein; Figure S4: Multiple sequence alignment related to the envelope protein; Figure S5: Multiple sequence alignment related to the membrane protein; Figure S6: Multiple sequence alignment related to the spike protein; Table S1: Coronavirus species considered in this study; Table S2: Dataset of 142 PTMs.
