# Supplementary material for: Evolutionary Signals in Coronaviral Structural Proteins Suggest Possible Complex Mechanisms of Post-Translational Regulation in SARS-CoV-2 Virus
Source: Viruses. 2022 Nov 8;14(11):2469. doi: 10.3390/v14112469 (PMC9696223; doi:10.3390/v14112469)
Supplement: Supplementary file 1 [file viruses-14-02469-s001.zip › Supplementary_Figure_S1.pdf]

(A)

Species	Position	Residue	PTM
Murine coronavirus	197	S	Phosphorylation
Severe acute respiratory syndrome-related coronavirus	177	S	Phosphorylation
Severe acute respiratory syndrome-related coronavirus	189	S	Phosphorylation
Severe acute respiratory syndrome coronavirus 2	184	S	Phosphorylation

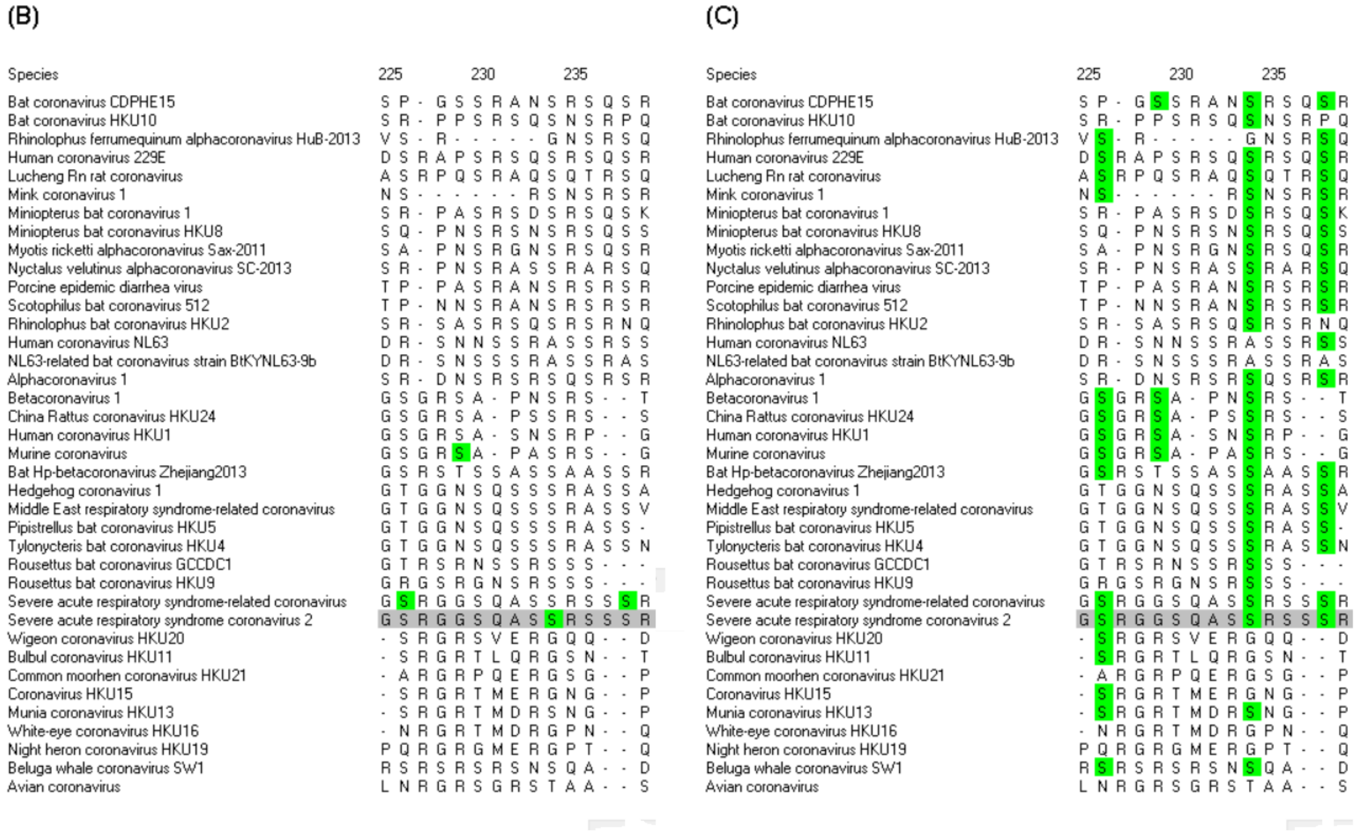

(D)

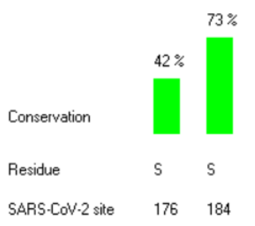

(E)

Residue	S	S	S
Position	176	184	188
Substitutions	9	8	9
Frequencies	R 20<br>G 17<br>I 15<br>N 15<br>T 5<br>C 2<br>P 1<br>L 1<br>M 1	P 70<br>F 57<br>Y 20<br>A 19<br>L 6<br>T 3<br>C 3<br>Q 1	L 1272<br>P 168<br>A 9<br>T 3<br>G 2<br>V 2<br>W 1<br>Q 1<br>F 1
Sum of frequencies	77	179	1459
Mutation frequency	0.052775	0.122686	1

Supplementary Figure S1. PTM Mapping, Propagation, Conservation and Mutation Frequency. (A) Illustrative dataset of 4 PTM sites. (B) Mapping the PTMs consists of identifying the positions of the PTMs on the alignment, PTM sites are represented in green. (C) Propagating a PTM refers to inferring that the modification exists in all species in which the residue is the same as the amino acid in the PTM site. (D) Degree of conservation of the PTM sites related to SARS-CoV-2 virus located in columns without gaps. (E) Frequencies of the different substitutions reported for the PTM sites, the sum of frequencies was normalized with respect to the higher value, we call this the mutation frequency for a PTM site.
