# Supplementary material for: Evolutionary Signals in Coronaviral Structural Proteins Suggest Possible Complex Mechanisms of Post-Translational Regulation in SARS-CoV-2 Virus
Source: Viruses. 2022 Nov 8;14(11):2469. doi: 10.3390/v14112469 (PMC9696223; doi:10.3390/v14112469)
Supplement: Supplementary file 1 [file viruses-14-02469-s001.zip › Supplementary_Figure_S2.pdf]

(A)

Position	Residue	PTM	Conservation (%)
47	N	N-Glycosylation	21
61	K	Sumoylation	52
76	T	Phosphorylation	10
78	S	Phosphorylation	15
105	S	Phosphorylation	7
141	T	Phosphorylation	10
148	T	O-Glycosylation	47
176	S	Phosphorylation	42
184	S	Phosphorylation	73
269	N	N-Glycosylation	39

(B)

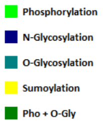

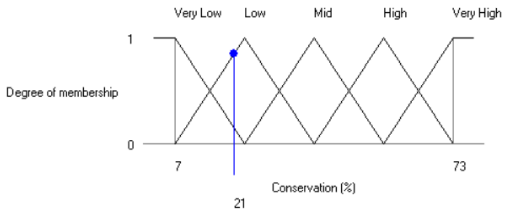

Fuzzy Sets	Degree of membership
Very Low	0.1515
Low	0.8484
Mid	0
High	0
Very High	0

(C)

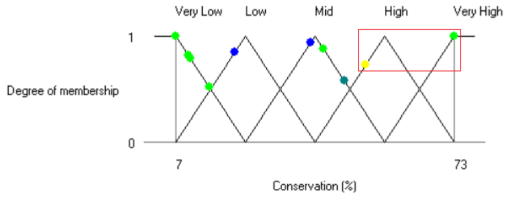

Position	Residue	PTM	Conservation (%)
47	N	N-Glycosylation	Low
61	K	Sumoylation	High
76	T	Phosphorylation	Very Low
78	S	Phosphorylation	Very Low
105	S	Phosphorylation	Very Low
141	T	Phosphorylation	Very Low
148	T	O-Glycosylation	Mid
176	S	Phosphorylation	Mid
184	S	Phosphorylation	Very High
269	N	N-Glycosylation	Mid

(D)

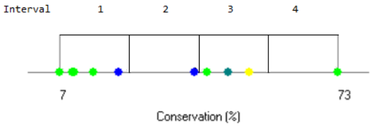

(E)

Position	Residue	PTM	Mutation Frequency
2	S	Phosphorylation	0.0871
23	S	Phosphorylation	0.0187
24	T	Phosphorylation	0.0500
26	S	Phosphorylation	0.0114
47	N	N-Glycosylation	0.0015
61	K	Sumoylation	0.0020
76	T	Phosphorylation	0.0020
78	S	Phosphorylation	0.0021
79	S	Phosphorylation	0.0071
105	S	Phosphorylation	0.0016
141	T	Phosphorylation	0.0227
148	T	O-Glycosylation	0.0057
165	T	O-Glycosylation	0.0113
166	T	Pho + O-Gly	0.0129
176	S	Phosphorylation	0.0004
180	S	Phosphorylation	0.0142
183	S	Phosphorylation	0.0643
184	S	Phosphorylation	0.0010
188	S	Phosphorylation	0.0086
194	S	Phosphorylation	0.5907
197	S	Phosphorylation	0.0566
198	T	Phosphorylation	0.0098
201	S	Phosphorylation	0.0255
202	S	Phosphorylation	0.1703
205	T	Pho + O-Gly	1
206	S	Pho + O-Gly	0.0419
269	N	N-Glycosylation	0.0021
391	T	Pho + O-Gly	0.1068
393	T	Phosphorylation	0.0123
404	S	O-Glycosylation	0.0010
410	S	Phosphorylation	0.0042
412	S	Phosphorylation	0.1016

(F)

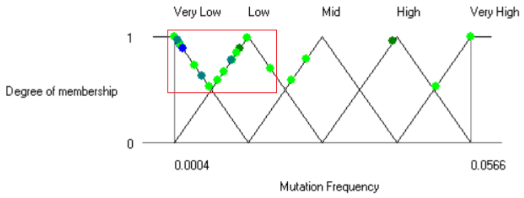

Position	Residue	PTM	Mutation Frequency	
23	S	Phosphorylation	0.0187	Low
24	T	Phosphorylation	0.0500	Very High
26	S	Phosphorylation	0.0114	Low
47	N	N-Glycosylation	0.0015	Very Low
76	T	Phosphorylation	0.0020	Very Low
78	S	Phosphorylation	0.0021	Very Low
79	S	Phosphorylation	0.0071	Very Low
105	S	Phosphorylation	0.0016	Very Low
141	T	Phosphorylation	0.0227	Mid
148	T	O-Glycosylation	0.0057	Very Low
165	T	O-Glycosylation	0.0113	Low
166	T	Pho + O-Gly	0.0129	Low
176	S	Phosphorylation	0.0004	Very Low
180	S	Phosphorylation	0.0142	Low
188	S	Phosphorylation	0.0086	Low
197	S	Phosphorylation	0.0566	Very High
198	T	Phosphorylation	0.0098	Low
201	S	Phosphorylation	0.0255	Mid
206	S	Pho + O-Gly	0.0419	High
269	N	N-Glycosylation	0.0021	Very Low
393	T	Phosphorylation	0.0123	Low
404	S	O-Glycosylation	0.0010	Very Low
410	S	Phosphorylation	0.0042	Very Low

Supplementary Figure S2. Selection process based on fuzzy sets. (A) Degree of conservation of the 10 PTM sites in the nucleocapsid protein related to SARS-CoV-2 virus located in columns without gaps. (B) Linguistic variable representing the degree of conservation, the variable is defined with a group of five overlapping fuzzy sets, and calculation of the degree of membership of the value 21 to the different fuzzy sets, see subsection 2.7 for explanations. (C) Fuzzification of the numeric values related to the degree of conservation, the PTM sites with linguistic values "High" and "Very High" (framed in a red rectangle) are considered sites of interest. (D) Selection process based on crisp intervals instead of fuzzy sets, see subsection 2.7 for details. (E) Values of the mutation frequency for the 32 PTM sites in the nucleocapsid protein. (F) Selection process of the PTM sites with the lowest mutation frequency, the PTM sites of interest are those with linguistic values "Very Low" and "Low" (framed in a red rectangle), see subsection 2.7 for explanations. The PTMs are represented in different colors as indicated at the top right of the Figure.
