# Supplementary material for: Evolutionary Signals in Coronaviral Structural Proteins Suggest Possible Complex Mechanisms of Post-Translational Regulation in SARS-CoV-2 Virus
Source: Viruses. 2022 Nov 8;14(11):2469. doi: 10.3390/v14112469 (PMC9696223; doi:10.3390/v14112469)
Supplement: Supplementary file 1 [file viruses-14-02469-s001.zip › Supplementary_Figure_S5.pdf]

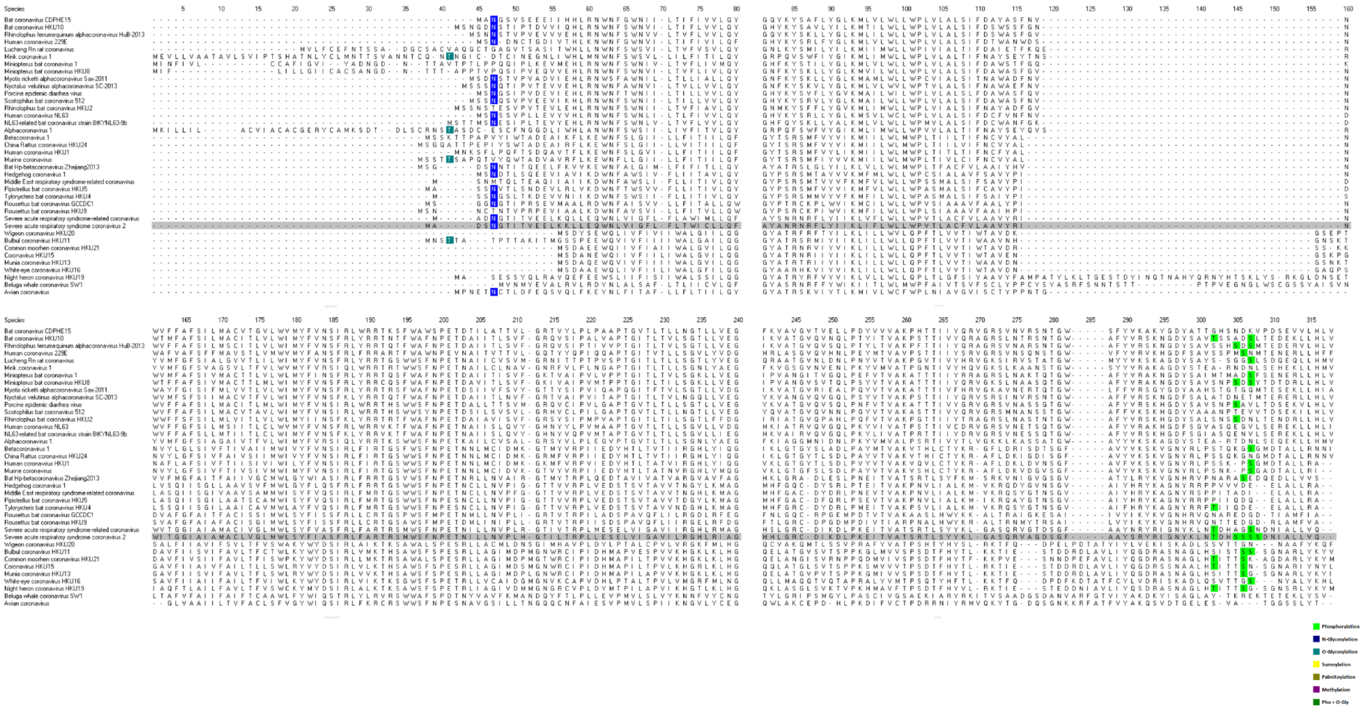

Supplementary Figure S5. Multiple sequence alignment related to the membrane protein. The numbers at the top correspond to the positions on the alignment. The species names of the 38 coronaviruses are listed on the left side, the row related to SARS-CoV-2 virus is highlighted in gray. The PTM sites are represented in different colors as indicated at the bottom right of the Figure.
