# Supplementary material for: Evolutionary Signals in Coronaviral Structural Proteins Suggest Possible Complex Mechanisms of Post-Translational Regulation in SARS-CoV-2 Virus
Source: Viruses. 2022 Nov 8;14(11):2469. doi: 10.3390/v14112469 (PMC9696223; doi:10.3390/v14112469)
Supplement: Supplementary file 1 [file viruses-14-02469-s001.zip › Supplementary_Figure_S6.pdf]

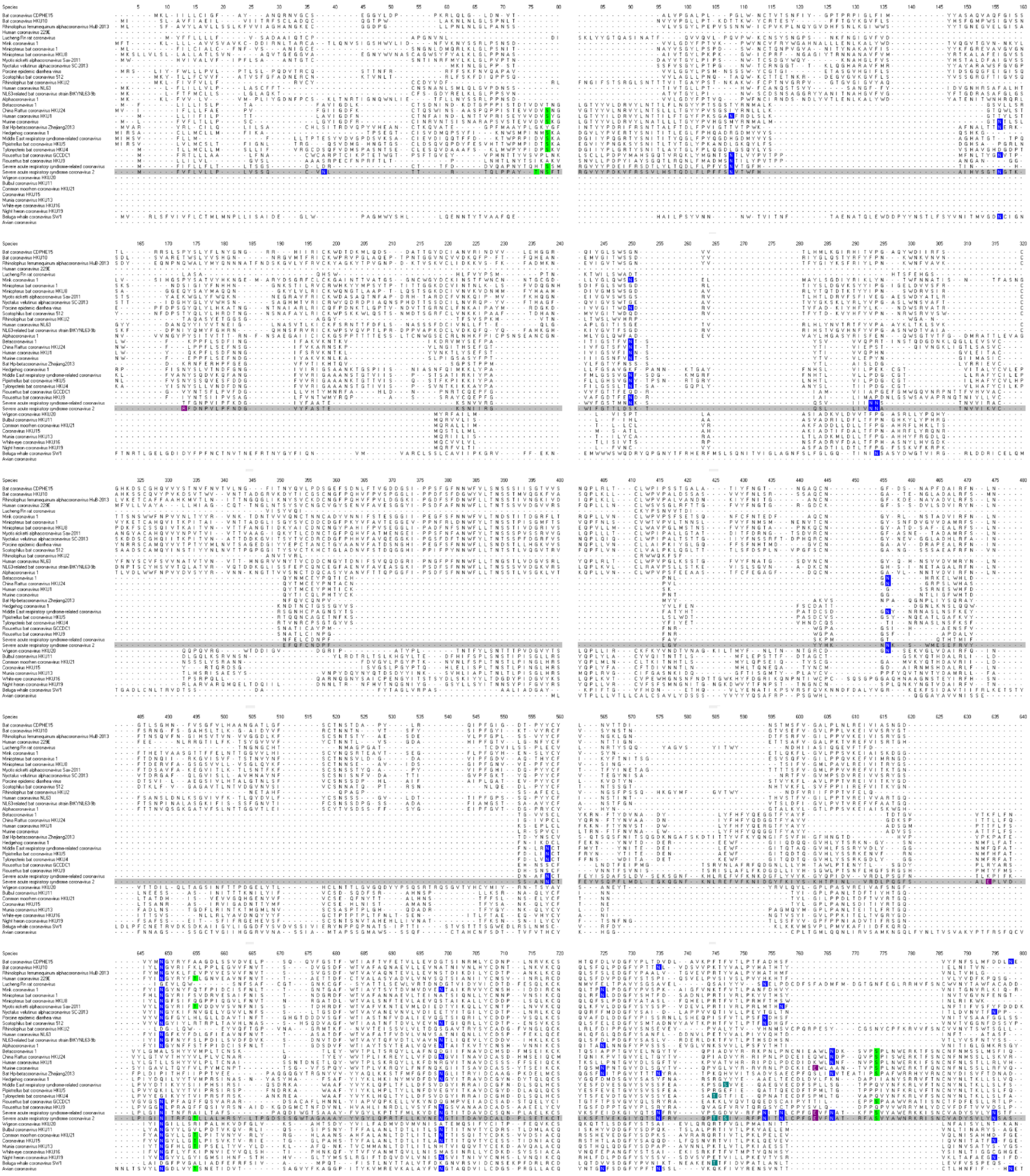

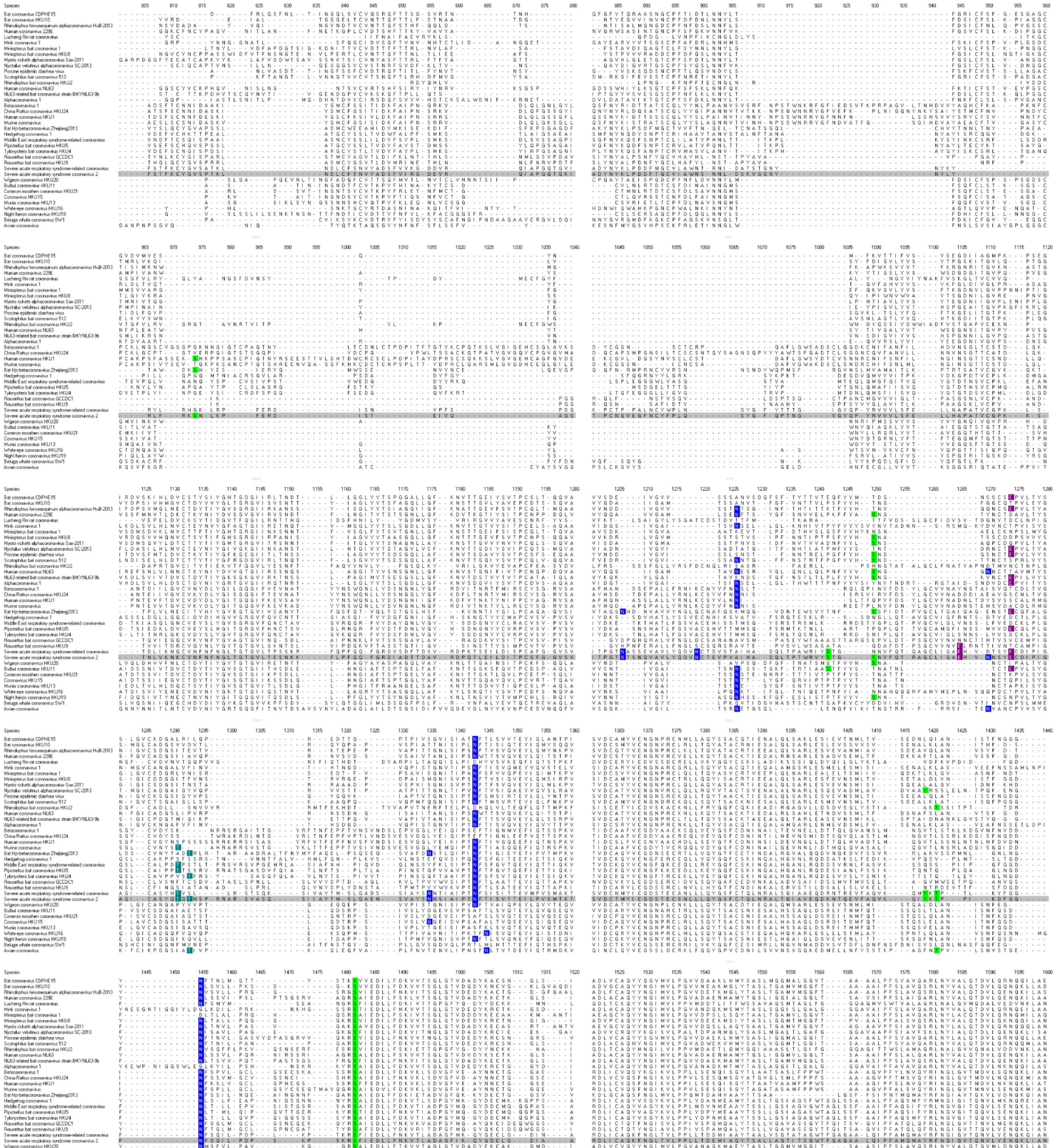
Species
Bat coronavirus CDPHE15
Bat coronavirus HKU10
Rhinolophus ferrumequinum alphacoronavirus HuB-2013
Human coronavirus 229E
Lucheng Rn rat coronavirus
Mink coronavirus 1
Miniopterus bat coronavirus 1
Miniopterus bat coronavirus HKU8
Myotis ricketti alphacoronavirus Sax-2011
Nyctalus velutinus alphacoronavirus SC-2013
Porcine epidemic diarrhea virus
Scotophilus bat coronavirus 512
Rhinolophus bat coronavirus HKU2
Human coronavirus NL63
NL63-related bat coronavirus strain BtKYNL63-9b
Alphacoronavirus 1
Betacoronavirus 1
China Rattus coronavirus HKU24
Human coronavirus HKU1
Murine coronavirus
Bat Hp-betacoronavirus Zhejiang2013
Hedgehog coronavirus 1
Middle East respiratory syndrome-related coronavirus
Pipistrellus bat coronavirus HKU5
Tylonycteris bat coronavirus HKU4
Rousettus bat coronavirus GCCDC1
Rousettus bat coronavirus HKU9
Severe acute respiratory syndrome-related coronavirus
Severe acute respiratory syndrome coronavirus 2
Wigeon coronavirus HKU20
Bulbul coronavirus HKU11
Common moorhen coronavirus HKU21
Coronavirus HKU15
Munia coronavirus HKU13
White-eye coronavirus HKU16
Night heron coronavirus HKU19
Beluga whale coronavirus SW1
Avian coronavirus

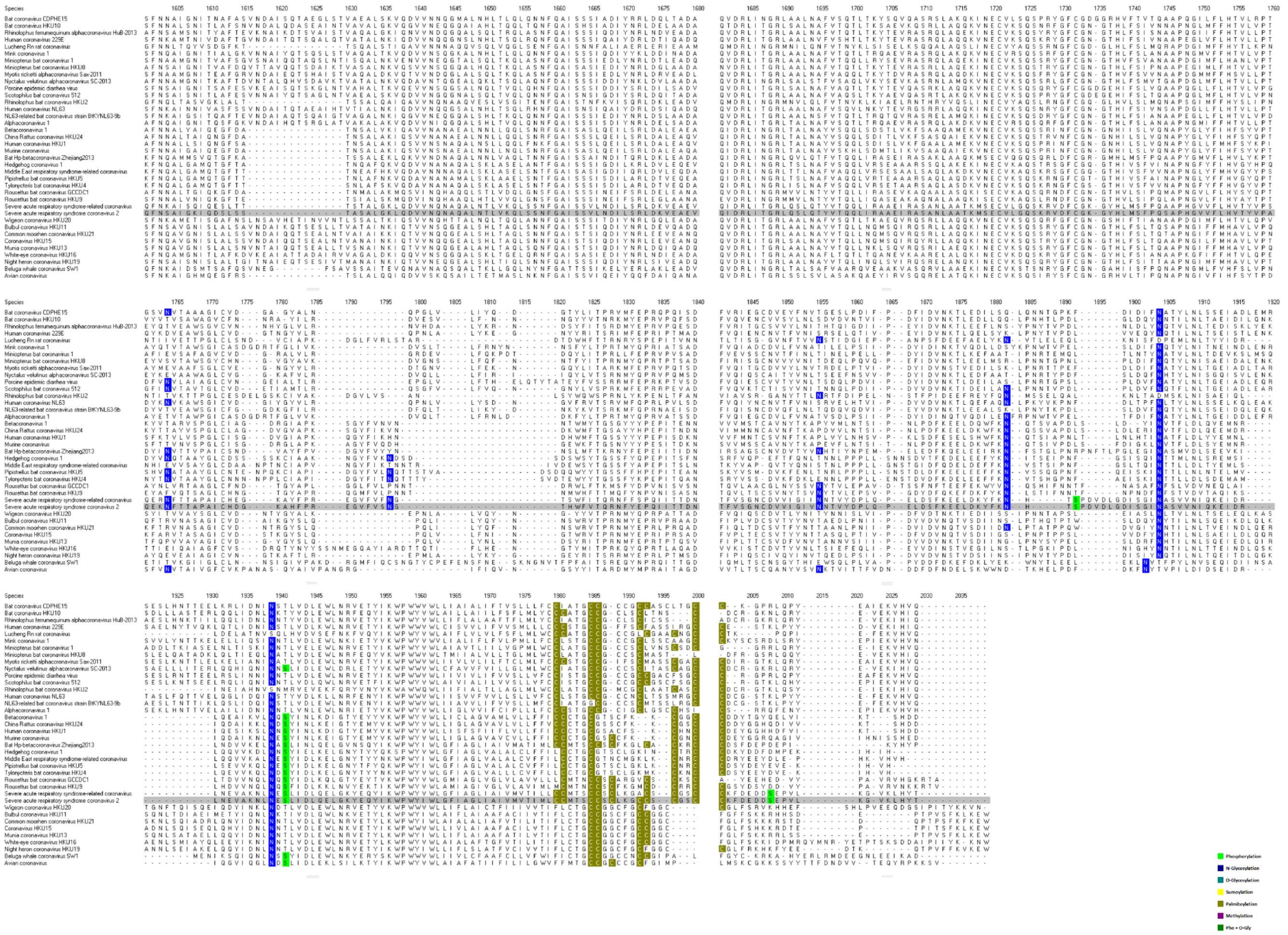

Supplementary Figure S6. Multiple sequence alignment related to the spike protein. The numbers at the top correspond to the positions on the alignment. The species names of the 38 coronaviruses are listed on the left side, the row related to SARS-CoV-2 virus is highlighted in gray. The PTM sites are represented in different colors as indicated at the bottom right of the Figure.
