# Supplementary material for: Evolutionary Signals in Coronaviral Structural Proteins Suggest Possible Complex Mechanisms of Post-Translational Regulation in SARS-CoV-2 Virus
Source: Viruses. 2022 Nov 8;14(11):2469. doi: 10.3390/v14112469 (PMC9696223; doi:10.3390/v14112469)
Supplement: Supplementary file 1 [file viruses-14-02469-s001.zip › Supplementary_Table_S1.pdf]

Supplementary Table S1. Coronavirus species considered in this study. The RefSeq accession numbers, genera, subgenera and species names of the 38 coronaviruses are listed in the table.

RefSeq	Genus	Subgenus	Species
NC_022103.1	Alphacoronavirus	Colacovirus	Bat coronavirus CDPHE15
NC_018871.1	Alphacoronavirus	Decacovirus	Bat coronavirus HKU10
NC_028814.1	Alphacoronavirus	Decacovirus	Rhinolophus ferrumequinum alphacoronavirus HuB-2013
NC_002645.1	Alphacoronavirus	Duvinacovirus	Human coronavirus 229E
NC_032730.1	Alphacoronavirus	Luchacovirus	Lucheng Rn rat coronavirus
NC_023760.1	Alphacoronavirus	Minacovirus	Mink coronavirus 1
NC_010437.1	Alphacoronavirus	Minunacovirus	Miniopterus bat coronavirus 1
NC_010438.1	Alphacoronavirus	Minunacovirus	Miniopterus bat coronavirus HKU8
NC_028811.1	Alphacoronavirus	Myotacovirus	Myotis ricketti alphacoronavirus Sax-2011
NC_028833.1	Alphacoronavirus	Nyctacovirus	Nyctalus velutinus alphacoronavirus SC-2013
NC_003436.1	Alphacoronavirus	Pedacovirus	Porcine epidemic diarrhea virus
NC_009657.1	Alphacoronavirus	Pedacovirus	Scotophilus bat coronavirus 512
NC_009988.1	Alphacoronavirus	Rhinacovirus	Rhinolophus bat coronavirus HKU2
NC_005831.2	Alphacoronavirus	Setracovirus	Human coronavirus NL63
NC_032107.1	Alphacoronavirus	Setracovirus	NL63-related bat coronavirus strain BtKYNL63-9b
NC_038861.1	Alphacoronavirus	Tegacovirus	Alphacoronavirus 1
NC_006213.1	Betacoronavirus	Embecovirus	Betacoronavirus 1
NC_026011.1	Betacoronavirus	Embecovirus	China Rattus coronavirus HKU24
NC_006577.2	Betacoronavirus	Embecovirus	Human coronavirus HKU1
NC_012936.1	Betacoronavirus	Embecovirus	Murine coronavirus
NC_025217.1	Betacoronavirus	Hibecovirus	Bat Hp-betacoronavirus Zhejiang2013
NC_039207.1	Betacoronavirus	Merbecovirus	Hedgehog coronavirus 1
NC_038294.1	Betacoronavirus	Merbecovirus	Middle East respiratory syndrome-related coronavirus
NC_009020.1	Betacoronavirus	Merbecovirus	Pipistrellus bat coronavirus HKU5
NC_009019.1	Betacoronavirus	Merbecovirus	Tylonycteris bat coronavirus HKU4
NC_030886.1	Betacoronavirus	Nobecovirus	Rousettus bat coronavirus GCCDC1
NC_009021.1	Betacoronavirus	Nobecovirus	Rousettus bat coronavirus HKU9
NC_004718.3	Betacoronavirus	Sarbecovirus	Severe acute respiratory syndrome-related coronavirus
NC_045512.2	Betacoronavirus	Sarbecovirus	Severe acute respiratory syndrome coronavirus 2
NC_016995.1	Deltacoronavirus	Andecovirus	Wigeon coronavirus HKU20
NC_011547.1	Deltacoronavirus	Buldecovirus	Bulbul coronavirus HKU11
NC_016996.1	Deltacoronavirus	Buldecovirus	Common moorhen coronavirus HKU21
NC_039208.1	Deltacoronavirus	Buldecovirus	Coronavirus HKU15
NC_011550.1	Deltacoronavirus	Buldecovirus	Munia coronavirus HKU13
NC_016991.1	Deltacoronavirus	Buldecovirus	White-eye coronavirus HKU16
NC_016994.1	Deltacoronavirus	Herdecovirus	Night heron coronavirus HKU19
NC_010646.1	Gammacoronavirus	Cegacovirus	Beluga whale coronavirus SW1
NC_001451.1	Gammacoronavirus	Igacovirus	Avian coronavirus
