# Supplementary material for: Evolutionary Signals in Coronaviral Structural Proteins Suggest Possible Complex Mechanisms of Post-Translational Regulation in SARS-CoV-2 Virus
Source: Viruses. 2022 Nov 8;14(11):2469. doi: 10.3390/v14112469 (PMC9696223; doi:10.3390/v14112469)
Supplement: Supplementary file 1 [file viruses-14-02469-s001.zip › Supplementary_Table_S2.pdf]

Supplementary Table S2. Dataset of 142 PTMs. PTM sites are listed according to the species, protein and PTM type, the corresponding references are shown in the last column.

Species	Protein	PTM	Sites	References
Alphacoronavirus 1	N	Phosphorylation	S9, S156, S254, S256	[17]
Murine coronavirus	E	Palmitoylation	C40, C44, C47	[18,19]
	M	O-Glycosylation	T5	[20]
	N	Phosphorylation	S170, T177, S197	[21,22]
Severe acute respiratory syndrome-related coronavirus	S	N-Glycosylation	N109, N118, N119, N158, N227, N318, N330, N357, N589, N699	[23,24,25]
			N783, N1056, N1080, N1140, N1155, N1176	[23,24,25]
	E	N-Glycosylation	N66	[26]
	E	Palmitoylation	C40, C43, C44	[27]
	M	N-Glycosylation	N4	[28]
	N	Phosphorylation	S177, S189, S207	[22,29]
	N	Sumoylation	K62	[30]
Severe acute respiratory syndrome coronavirus 2	S	N-Glycosylation	N17, N61, N74, N122, N149, N165, N234, N282, N331, N334	[31-35]
			N343, N603, N616, N657, N709, N717, N801, N1074, N1098, N1134	[31-35]
			N1158, N1173, N1194	[31-35]
	S	O-Glycosylation	T323, S325, T676, T678	[33,36,37]
	S	Methylation	R78, E224, E340, E654, E661	[35]
	S	Palmitoylation	C1235, C1236, C1240, C1241, C1243, C1247, C1248, C1250, C1253, C1254	[38]
	S	Phosphorylation	T29, S31, T240, S349, S459, S637, S640, Y789, T791, S816	[39]
			S1161, S1196, S1261	[39]
	M	Phosphorylation	T208, S211, S212, S213, S214	[39,40,41]
	N	Phosphorylation	S2, S23, T24, S26, T76, S78, S79, S105, T141, T166	[39,40,41,42]
			S176, S180, S183, S184, S194, S197, T198, S201, S202, T205	[39,40,41,42]
			S206, T391, T393, S410, S412	[39,40,41,42]
	N	N-Glycosylation	N47, N269	[42]
	N	O-Glycosylation	T148, T165, T166, T205, S206, T391, S404	[42]
Avian coronavirus	S	N-Glycosylation	N212, N237, N247, N276, N513, N591, N1051, N1074	[43]
	N	Phosphorylation	S190, S192, T378, S379	[44]
